# Central Sensitisation After Orthopaedic Trauma: An Overlooked Contributor to Chronic Pain and Functional Disability—A Scoping Review

**DOI:** 10.3390/jcm15031035

**Published:** 2026-01-28

**Authors:** Arfaz Shaik, Arjun Chakrapani, Aaron Alexander, Abdullah Al Jumaili, Umar Hayat

**Affiliations:** 1 Department of Trauma and Orthopaedics, Croydon Health Services NHS Trust, London CR7 7YE, UK; 2 Department of Trauma and Orthopaedics, Torbay and South Devon NHS Trust, Torquay TQ2 7AA, UK; 3 Department of Trauma and Orthopaedics, University Hospital Llandough, Cardiff CF64 2XX, UK; 4 Department of Trauma and Orthopaedics, Medway Maritime Hospital, Medway NHS Foundation Trust, Gillingham ME7 5NY, UK

**Keywords:** central sensitisation, orthopaedic trauma, chronic post-traumatic pain, neuropathic pain, pain chronification, psychological distress, widespread pain, functional disability, scoping review

## Abstract

**Background:** Persistent pain following orthopaedic trauma is common, often disproportionate to structural healing, and increasingly interpreted as reflecting centrally mediated pain mechanisms. However, the mechanisms, clinical features, diagnostic approaches, prognostic indicators, and management strategies relevant to trauma-related central sensitisation (CS) remain poorly understood. **Objective:** To map and synthesise existing evidence on CS following orthopaedic trauma, addressing mechanistic pathways, clinical manifestations, epidemiology, assessment methods, management approaches, and health system implications. **Methods:** A scoping review was conducted in accordance with PRISMA-ScR. Twenty-one studies met the eligibility criteria, comprising nine primary trauma cohorts and 12 contextual mechanistic or review studies relevant to trauma-associated CS. Data were charted across six prespecified domains of mechanistic processes, clinical presentation and diagnostic features, epidemiology and prognosis, assessment tools and outcome measures, interventions, and health system and care delivery considerations. **Results:** Mechanistic studies demonstrated trauma-induced neuroimmune activation, altered cortical and spinal excitability, and molecular pathways consistent with sensitisation. Clinical studies have identified neuropathic features, widespread pain, and heightened sensory responsiveness following fractures and other injuries. Neurophysiological evidence has indicated early cortical disinhibition following upper limb trauma, whereas epidemiological cohorts have reported persistent pain and disability years after major trauma. Measurement studies have highlighted the limited reliability and specificity of current tools in trauma populations, including quantitative sensory testing and self-report instruments. Early predictors of adverse trajectories include severe acute pain, neuropathic descriptors, psychological distress, and opioid-dominant analgesia. Evidence regarding early intervention, rehabilitation strategies, and system-level screening pathways remains limited. **Conclusions:** Central sensitisation (CS)–consistent mechanisms after orthopaedic trauma are suggested by convergent mechanistic, neurophysiological, and clinical findings. However, trauma-specific diagnostic criteria, prognostic models, and management frameworks remain underdeveloped. High-quality longitudinal research is needed to clarify early trajectories, refine assessment methods, and establish targeted interventions to reduce long-term pain and disability.

## 1. Introduction

Orthopaedic trauma is a major cause of long-term pain and disability worldwide. Despite satisfactory structural healing, a substantial proportion of patients develop persistent symptoms that cannot be fully explained by ongoing tissue injury or identifiable pathology [1,2]. Chronic post-traumatic pain commonly presents with sensory amplification, disproportionate symptom intensity, and widespread hypersensitivity, features suggestive of centrally mediated mechanisms.

A clear conceptual distinction between pain mechanisms is essential in trauma populations. Nociception refers to the normal neural processes by which potentially harmful stimuli are detected and transmitted to the central nervous system [3]. Nociceptive pain arises directly from this process in the presence of ongoing tissue injury or inflammation [3,4]. Neuropathic pain is defined as pain caused by a demonstrable lesion or disease of the somatosensory nervous system, such as traumatic nerve damage [3,5]. Nociplastic pain describes pain that persists despite minimal or resolved peripheral pathology and is attributed to altered nociceptive processing without clear structural or neural injury [3,6]. Central sensitisation (CS), in contrast, is not a diagnostic category but a neurophysiological process characterised by amplified central pain signalling driven by altered inhibitory–excitatory balance, neuroimmune activation, and synaptic plasticity [3,7,8,9]. CS can contribute to nociplastic pain and to the amplification of both nociceptive and neuropathic pain states [6,10]. Accordingly, in this review, CS is treated as a unifying explanatory mechanism for disproportionate and persistent post-traumatic pain rather than as a discrete clinical diagnosis.

Traumatic musculoskeletal injuries differ fundamentally from controlled surgical procedures. High-energy mechanisms, crush forces, open fractures, and extensive soft-tissue damage generate intense and prolonged nociceptive input that is more variable and severe than that seen in elective operative settings [7,11]. Whereas elective surgery typically involves predictable tissue handling and a relatively uniform inflammatory response, trauma provokes heterogeneous biological cascades—including chemokine signalling, microglial activation, glial proliferation, and cytokine release—that are implicated in sustained pain hypersensitivity [8,9]. Clinical studies support early central involvement after trauma: disruption of intracortical inhibition has been demonstrated within two weeks of fracture [12], and severe acute pain is strongly associated with later persistent pain [13].

Identifying CS after trauma remains challenging. Post-traumatic pain often exhibits overlapping nociceptive and neuropathic features and may be influenced by immobilisation, nerve irritation, surgical revision, psychological distress, and social factors, making attribution to a single mechanism difficult [2,14,15]. Assessment tools such as the Central Sensitisation Inventory (CSI), Quantitative Sensory Testing (QST), PainDETECT, and the Douleur Neuropathique (DN4) questionnaire have been evaluated in musculoskeletal pain, but their measurement properties are inconsistent in trauma cohorts [16,17]. Widespread pain, frequently used as a surrogate marker of central amplification, may reflect psychological distress as much as biological sensitisation [14,18].

Epidemiological evidence indicates that a substantial subgroup of trauma patients progress to chronic pain with centrally mediated features. Neuropathic pain descriptors are reported in up to one-third of patients following major lower-limb trauma and are associated with greater disability and poorer quality of life [2]. Longitudinal studies demonstrate that trauma exposure increases the risk of new-onset widespread pain [18], and chronic pain remains common even when structural healing is satisfactory [15]. Early predictors—including severe acute pain, high opioid exposure without multimodal strategies, catastrophising, sleep disturbance, and PTSD symptoms—have been linked to adverse long-term outcomes [11,13,14]. However, the mechanisms connecting these early factors to chronic centrally mediated pain remain unclear.

Management strategies targeting central mechanisms have been inconsistently applied to trauma practices. Although multimodal analgesia can reduce nociceptive input and attenuate early sensitisation, its integration into acute trauma pathways remains variable [11,19]. Psychological distress and PTSD, which are prevalent after trauma, can exacerbate centrally mediated pain, yet structured screening and targeted interventions are rarely embedded in routine orthopaedic practice [14]. Rehabilitation strategies aimed at promoting early loading and movement may inadvertently worsen symptoms in patients with sensory hyperexcitability or fear-avoidant behaviours [11]. System-level gaps include limited recognition of CS in trauma clinics, the absence of consensus outcome measures, and the lack of integration of CS assessment into fracture care pathways.

Collectively, these uncertainties highlight the need for a comprehensive synthesis of evidence specific to CS after orthopaedic trauma. In this review, CS is treated as a mechanistic construct rather than a discrete clinical diagnosis. Because validated trauma-specific diagnostic criteria do not currently exist, CS in orthopaedic trauma populations is inferred from convergent features, including disproportionate and persistent pain, neuropathic pain characteristics, altered sensory processing, psychological vulnerability, and functional impairment. Importantly, the presence of persistent or neuropathic pain alone is not equated with CS; instead, these features are interpreted as proxy indicators of centrally mediated pain mechanisms. Given the heterogeneity of study designs, outcome measures, and conceptual definitions of CS in trauma populations, a scoping review was chosen to map the breadth and nature of the evidence rather than to perform quantitative synthesis.

Accordingly, this scoping review aims to clarify the following:

1. How CS is conceptualised within orthopaedic trauma literature and how it identifies areas of definitional inconsistency.
2. Describe how CS-related symptoms, diagnostic tools, and early risk indicators are applied to or interpreted in injured populations.
3. Map the current evidence on assessment approaches, prognostic patterns, management strategies, and system-level implications relevant to CS after orthopaedic trauma.
4. Examining the extent to which existing evidence supports the hypothesis that early identification of CS after orthopaedic trauma might reduce chronic pain, unnecessary reoperations, and morbidity, while explicitly acknowledging where direct data are lacking.

## 2. Materials and Methods

### 2.1. Protocol and Registration

This review followed the methodological framework for scoping reviews and was reported in accordance with the PRISMA-ScR (Preferred Reporting Items for Systematic Reviews and Meta-Analyses extension for Scoping Reviews) guidelines. This scoping review protocol was prospectively registered on the Open Science Framework (OSF; Registration DOI: 10.17605/OSF.IO/A4NEP).

### 2.2. Eligibility Criteria

Eligibility criteria were established to capture evidence of CS specifically within the context of orthopaedic trauma, including the mechanistic, neurophysiological, clinical, epidemiological, assessment-related, psychological, and management domains of the disease.

Inclusion Criteria

- Population: Adults or adolescents with orthopaedic trauma, including fractures, dislocations, high-energy limb injuries, crush injuries, soft tissue injuries, or polytrauma.
- Concept: Studies were eligible if they assessed constructs consistent with centrally mediated pain mechanisms (e.g., neuropathic pain, widespread pain, pain chronification, cortical disinhibition, or psychological amplification), even if the term ‘central sensitisation’ was not explicitly used after orthopaedic trauma.
- Context: Acute, subacute, or chronic post-traumatic phases; inpatient, outpatient, surgical, or rehabilitation settings.
- Study designs: Mechanistic laboratory studies, neurophysiology studies, observational cohorts, prospective studies, clinical prediction studies, systematic reviews, and thematic or narrative reviews.
- Outcomes: Any outcome related to pain, sensory testing, neurophysiology, functional disability, psychological features, or long-term morbidity.

Exclusion Criteria

- Studies focusing solely on chronic non-traumatic pain
- Elective orthopaedic surgery without a trauma cohort, unless directly comparing traumatic vs. surgical mechanisms
- Animal studies
- Non-peer reviewed literature (conference abstracts, dissertations).

### 2.3. Information Sources

A comprehensive literature search was conducted across seven electronic databases: MEDLINE, Embase, Scopus, Web of Science, Cochrane Library, ProQuest, and Google Scholar.

### 2.4. Search Strategy

The search strategy combined controlled vocabulary (e.g., MeSH terms) with free-text keywords related to

- central sensitisation, nociplastic pain, pain modulation, widespread pain, quantitative sensory testing, cortical excitability, temporal summation, conditioned pain modulation, combined with
- trauma, fracture, orthopaedic trauma, musculoskeletal injury, post-traumatic, post-surgical, lower limb fracture, upper limb fracture.

An example MEDLINE search string is as follows: (fracture* OR “musculoskeletal injury” OR “orthopaedic trauma” OR trauma* OR polytrauma) AND (“central sensitization” OR “central sensitisation” OR nociplastic OR hyperalgesia OR allodynia OR “cortical excitability”) AND (pain OR neuropathic OR persistent OR chronic).

### 2.5. Selection of Sources of Evidence

The search results were imported into a citation management system, and duplicates were removed. Screening was performed in two stages:

1. Title and abstract screening. 2. Full-text eligibility assessment.

Two reviewers independently screened the abstracts and full texts. Disagreements were resolved by discussion. The PRISMA flow diagram illustrates the screening and selection process.

Twenty-one sources of evidence were included, comprising nine primary trauma cohorts and 12 contextual mechanistic or review studies.

### 2.6. Data Charting Process

A structured data-charting form was developed to extract the following information.

- Study design, sample, population characteristics
- Nature and mechanism of injury
- Mechanistic findings
- Neurophysiological markers
- Clinical features and pain phenotypes
- Psychological correlations
- QST or measurement outcomes
- Treatment or analgesic findings
- Prognostic outcomes and trajectory findings

Data were charted independently by two reviewers and compared for accuracy. Systematic, narrative, and mechanistic reviews were included to provide contextual interpretation of central sensitisation mechanisms, but were not used to estimate prevalence, effect sizes, or prognostic associations.

### 2.7. Synthesis of Results

Due to the variability in study designs and analytical approaches, we employed a narrative thematic synthesis. Meta-analysis was not feasible due to the substantial clinical, methodological, and conceptual heterogeneity across trauma types, CS constructs, outcome measures, and follow-up durations. In line with PRISMA-ScR guidelines, we did not conduct a formal risk-of-bias assessment, as our objective was to provide a structured overview of the available literature rather than to estimate pooled effects. Instead, we narratively considered study design, sample characteristics, outcome measures, and methodological limitations in our interpretation of the findings. The findings were organised according to the six domains defined a priori:

1. Mechanistic and Pathophysiological Processes
2. Clinical Manifestation and Diagnostic Features
3. Epidemiology and Prognosis
4. Assessment and Measurement
5. Intervention and Management
6. Health-System and Outcomes

This domain structure reflects the multidimensional nature of centrally mediated pain mechanisms after orthopaedic trauma and aligns with the established theoretical models of nociplastic and trauma-related pain [9,11,20].

## 3. Results

Across the included trauma cohorts, CS was rarely assessed using direct neurophysiological or sensory testing frameworks. Instead, centrally mediated pain mechanisms were inferred primarily through neuropathic pain features, pain persistence disproportionate to structural healing, altered sensory symptoms, psychological vulnerability, and, in one study, cortical excitability measured by transcranial magnetic stimulation. Accordingly, results are synthesised according to the type of CS-consistent proxy used rather than interpreted as definitive diagnoses of CS.

### 3.1. Study Selection

The search and screening process identified studies examining pain outcomes, sensory phenotypes, and centrally mediated mechanisms following orthopaedic trauma. After removing duplicates and applying predefined inclusion and exclusion criteria, 21 studies were retained for the qualitative synthesis. Nine were primary clinical studies involving trauma-exposed populations, and 12 provided contextual evidence to support the interpretation of central sensitisation.

**Figure 1:**
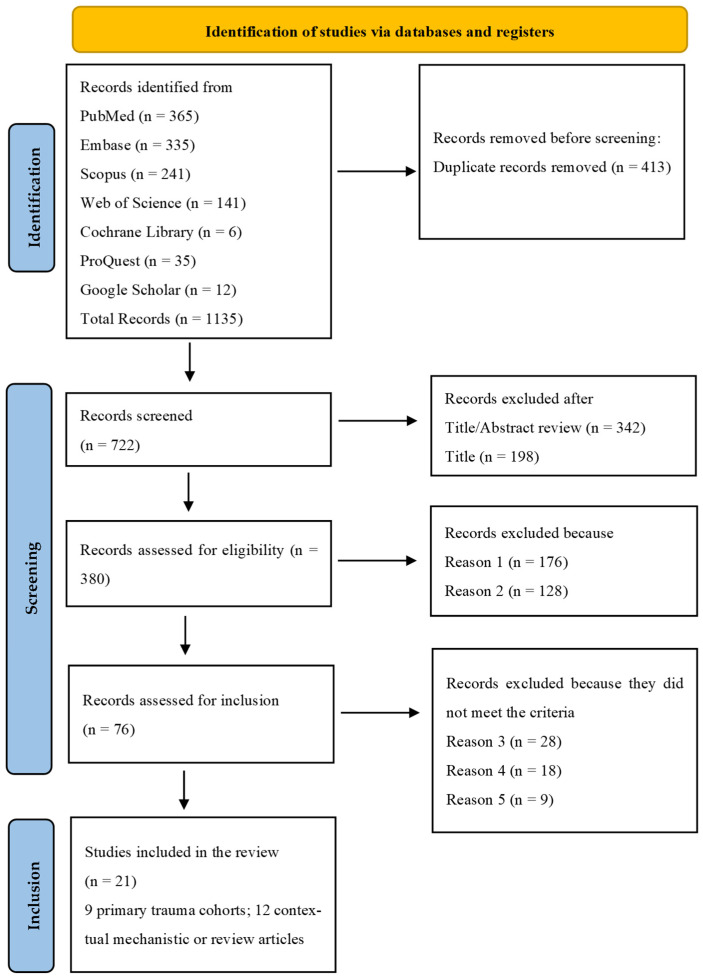
PRISMA-ScR flow diagram of the search and selection process for sources of evidence in this scoping review. Note: Reason 1: Sources not addressing central sensitisation (CS) mechanisms, prevalence, or impacts post-orthopaedic trauma (e.g., general chronic pain without trauma focus); Reason 2: Non-adult populations (<18 years) or non-orthopaedic contexts (e.g., neuropathic pain only); Reason 3: Full-text articles excluded after eligibility assessment because they did not report trauma-specific CS-related outcomes (e.g., no CS-relevant measures, no pain phenotype data, or no trauma subgroup analysis); Reason 4: Study type exclusion—case reports, narrative commentaries, editorials, or conference abstracts without extractable clinical or mechanistic data.; Reason 5: Publication date before 2000 or non-English language articles.

The nine primary studies included prospective cohorts [13,18,21], retrospective and cross-sectional outcome studies [1,22,23], neurophysiological experiments [12], and multicentre trauma trial datasets [2,24]. The contextual evidence comprised systematic reviews and meta-analyses [16,17,25,26], narrative and consensus reviews [7,8,11,15,19,27], and clinical or methodological studies addressing pain modulation [14,28]. These secondary sources were used solely for interpretive purposes and were not included in the PRISMA-ScR evidence map. The study selection process is summarised in Figure 1.

### 3.2. Characteristics of Included Trauma Cohorts

Large multicenter datasets included the UK WHiST trial cohort of surgically treated lower limb fractures [2] and a US multicenter extremity fracture randomised controlled trial [24]. Additional cohorts included patients undergoing fracture osteosynthesis [21], pelvic trauma [22], unstable ankle fractures [23], major blunt trauma [1], and acute isolated fractures evaluated using neurophysiological methods [12]. A national UK insurance cohort captured post-collision (MVC) musculoskeletal pain trajectories [18]. A prospective cohort study of a heterogeneous general orthopaedic trauma population examined predictors of severe acute pain and persistent post-surgical pain [13]. Follow-up periods ranged from days to more than six years.

No included study applied a formal diagnostic framework for central sensitisation. Instead, CS-consistent constructs were inferred from neuropathic pain features, neurophysiological changes, persistent pain, and psychological amplification [2,7,8,16,17]. Detailed study-level data extraction for the nine primary trauma cohorts is presented in Supplementary Table S1, while the twelve sources of secondary contextual evidence are summarised in Supplementary Table S2, and a concise overview of the nine primary cohorts is presented in Table 1.

#### 3.2.1. Outcome Measures and Methodological Heterogeneity

There was marked heterogeneity across the studies in how pain and potential CS were measured. Studies variably employed pain intensity scales (e.g., numerical rating scale or visual analogue scale) [1,13,21], neuropathic pain screening tools such as DN4, DN4i, painDETECT, and the McGill Pain Questionnaire [2,21,23], and multidimensional disability or quality-of-life instruments including EQ-5D, Disability Rating Index, SF-12, Oswestry Index, and fracture-specific outcome scores [2,21,22]. Objective or mechanistic measures were rarely used; only one study applied transcranial magnetic stimulation to evaluate intracortical inhibition and facilitation as proxies of central processing [12]. This methodological variability limits direct comparison across cohorts and precludes quantitative pooling and supports the need for standardised phenotype definitions in trauma pain research.

#### 3.2.2. Timing of Assessments

Assessments of CS-related outcomes occurred across distinct temporal windows:

- Acute (≤2 weeks): Objective neurophysiological changes associated with pain intensity were measured using TMS shortly after isolated fractures [12].
- Subacute (3–6 months): Most cohorts evaluated persistent pain, neuropathic features, and functional impact during early recovery [2,13,18,21].
- Chronic (≥12 months to years): Long-term studies documented enduring pain burden and neuropathic symptoms extending from 12 months to more than six years post-injury [1,21,22,23]. This spread of time points highlights that CS-consistent features can be detected from the immediate post-injury period through long-term follow-up.

### 3.3. Epidemiology and Prognostic Patterns

#### 3.3.1. Prevalence of CS-Related Symptoms After Trauma

Persistent pain and neuropathic pain features were common across all included trauma cohorts, with prevalence varying by injury type, severity, and duration of follow-up. Across studies, chronic pain prevalence after orthopaedic trauma ranged from 30–70% at 3–12 months, with neuropathic characteristics present in approximately one-quarter of patients [1,13,21,22].

In the UK WHiST multicenter cohort of surgically treated lower-limb fractures, approximately one-third of the patients reported neuropathic pain characteristics at three and six months post-injury, measured using validated descriptors of neuropathic pain [2]. Importantly, neuropathic features emerged in some patients over time, suggesting that centrally mediated pain phenotypes can develop during recovery rather than being confined to the early post-injury period [2].

Similar findings were observed in a German fracture osteosynthesis cohort, where chronic post-traumatic or post-surgical pain was prevalent at three and 12 months, and a substantial proportion of symptomatic patients screened positive for neuropathic pain using the DN4 and painDETECT screening tools [21]. Long-term studies further demonstrated that pain frequently persisted well beyond the expected tissue healing period. Among pelvic fracture survivors, 64% reported ongoing pain at a median of 52 months after injury, with many meeting higher grades of chronicity on the Mainz Pain Staging System [22]. In operatively treated unstable ankle fractures, 23% of patients fulfilled criteria for neuropathic pain at a mean follow-up of 5.8 years [23]. These figures demonstrate that neuropathic features remain detectable for many years after injury.

Similarly, in a multicentre cohort of major blunt trauma, nearly half of patients reported chronic pain six years after injury, with severity ranging from intermittent moderate pain to persistent severe pain interfering with daily activities [1]. Persistent symptoms beyond the initial injury region were also observed following musculoskeletal trauma: a national UK motor-vehicle collision cohort reported new-onset widespread pain in 8% of individuals at six months [18].

Across these diverse populations, neuropathic pain descriptors, long-term pain persistence, and associated functional interference were consistently observed, indicating that a substantial proportion of patients experience pain that is not fully explained by peripheral tissue recovery after orthopaedic trauma. Table 2 summarises pain phenotypes and their prevalence across studies.

#### 3.3.2. Predictors of Persistent Pain After Orthopaedic Trauma

Across the included trauma cohorts, the prognostic patterns were broadly consistent. In a major blunt trauma cohort with a six-year follow-up, the injury severity score (ISS) was the strongest predictor of chronic pain, independent of demographic factors [1]. Multicentre extremity fracture trial datasets similarly demonstrated substantial rates of persistent pain among patients with severe limb injuries, reinforcing the importance of injury magnitude and complexity as determinants of adverse outcomes [24].

Clinical evidence of nerve injury was associated with more advanced pain chronicity and greater symptom severity beyond skeletal healing. In pelvic fracture survivors, documented neural injury correlated with higher stages of pain chronicity and more severe persistent pain syndromes [22]. Long-term follow-up of unstable ankle fractures also identified a distinct subgroup of patients with neuropathic pain features, supporting the notion that peripheral neural disruption and adverse recovery trajectories may contribute to persistent pain [23].

Similarly, higher early pain intensity was consistently associated with later persistent pain/chronicity in trauma surgery and fracture-related surgery cohorts [13,21]. Psychological distress, prior analgesic use, and somatic symptom burden were repeatedly associated with chronic pain and poorer long-term outcomes [2,18]. Finally, female sex was associated with severe acute pain in immediate postoperative recovery in one cohort [13]. Although no study formally diagnosed central sensitisation, these factors collectively characterised patients who developed persistent pain with neuropathic features and disability, a pattern consistent with susceptibility to centrally mediated pain states.

#### 3.3.3. Objective Evidence of Central Nervous System Change After Orthopaedic Trauma

Only one included study directly assessed central nervous system function using neurophysiological methods. Jodoin et al. [12] examined motor cortex excitability using transcranial magnetic stimulation (TMS) in patients with acute upper limb fractures within the first two weeks following injury. Patients reporting moderate to severe pain (numerical rating scale ≥ 4) demonstrated significant reductions in short interval intracortical inhibition (SICI) and intracortical facilitation (ICF) compared to healthy controls and trauma patients with lower pain intensity. These findings indicate an early disruption of the normal inhibitory–excitatory balance within the primary motor cortex, which is consistent with cortical disinhibition and central hyperexcitability.

Importantly, these neurophysiological abnormalities were observed during the acute post-injury period, prior to the development of chronic pain. The magnitude of cortical disinhibition was proportional to the reported pain intensity, supporting a dose–response relationship between nociceptive load and central nervous system adaptation.

Although derived from a single cohort, this study provides direct neurophysiological evidence linking acute traumatic pain to early alterations in central processing that are compatible with the centrally mediated pain phenotypes observed in longer-term trauma cohorts.

Indirect clinical evidence supporting CS included: (i) high rates of persistent pain that were not confined to immediate postoperative periods [13,21], (ii) substantial proportions of patients reporting neuropathic characteristics after fracture surgery [2,23], and (iii) the occurrence of new-onset widespread pain after crash in a subgroup, with psychosocial factors influencing risk [18].

Collectively, these data support a trajectory in which early high-intensity pain and neurophysiological alterations may precede longer-term mixed phenotypes (nociceptive–neuropathic–centralised), though the included studies rarely measured the same central sensitisation constructs longitudinally within the same cohort.

#### 3.3.4. Psychological and Behavioural Modifiers of Pain Persistence After Orthopaedic Trauma

Psychological distress and maladaptive cognitive–affective responses were consistently associated with worse pain outcomes after orthopaedic trauma. In a prospective cohort of general orthopaedic trauma surgery patients, Edgley et al. [13] reported that higher perioperative pain catastrophising and psychological distress were linked to greater acute pain severity and an increased risk of persistent post-surgical pain and disability. These associations remained evident even after adjustment for clinical and surgical factors, indicating that psychological vulnerability contributes meaningfully to pain persistence and functional interference.

Similar findings were observed in the fracture-specific cohort. Aulenkamp et al. [21] demonstrated that patients with chronic post-traumatic pain, particularly those with neuropathic pain features, reported higher levels of anxiety and depressive symptoms at follow-up, supporting an interaction between affective distress, psychological vulnerability, and pain chronification. Evidence from motor-vehicle collision–related musculoskeletal injuries similarly indicates that recovery trajectories are heterogeneous and influenced by non-structural factors. This cohort also showed that long-term pain and functional limitations were not fully explained by the severity of physical injury, highlighting the influence of psychosocial and behavioural factors on recovery trajectories [18].

Collectively, these studies indicate that psychological and behavioural factors are closely linked to pain persistence and disability after trauma and help identify patient subgroups at increased risk of developing centrally mediated pain states.

### 3.4. Long-Term Outcome Burden and Functional Impact After Orthopaedic Trauma

Across primary trauma cohorts, persistent pain was consistently associated with clinically meaningful disability and reduced health-related quality of life, often extending years beyond the index injury. In a major blunt trauma cohort with six-year follow-up, chronic pain frequently interfered with daily activities, ranging from intermittent moderate symptoms to persistent severe pain. Importantly, these limitations were observed even in the absence of ongoing structural pathology, indicating that functional impairment can remain prominent after completion of standard trauma care [1].

Similar associations were seen in pelvic fracture survivors, where higher pain chronicity stages correlated with worse disability and poorer physical quality-of-life scores several years after injury [22].

Functional impact was also evident after more localised injuries. In unstable ankle fracture cohorts, patients with persistent or neuropathic pain features reported greater activity limitation and lower quality of life at long-term follow-up [23]. At earlier stages of recovery, both the WHiST cohort and fracture osteosynthesis studies demonstrated that neuropathic or persistent pain was strongly associated with greater disability, impaired rehabilitation, higher pain interference, and poorer patient-reported health status [2,21].

Collectively, these findings demonstrate that persistent and neuropathic pain phenotypes after orthopaedic trauma translate into sustained functional impairment and reduced quality of life, with measurable impacts from early recovery through multi-year follow-up.

### 3.5. Health-System and Outcome Implications

Persistent pain after orthopaedic trauma represents a substantial and often under-recognised burden for health systems. Multiple prospective cohorts demonstrate that moderate-to-severe pain remains highly prevalent months to years after injury, despite successful fracture healing [1,13,21,22]. This persistent pain is frequently neuropathic or centralised in nature rather than purely nociceptive, with neuropathic features reported in 17–32% of patients following lower limb or ankle fracture surgery [2,23]. Failure to recognise these mechanisms may result in repeated consultations, prolonged opioid use, and potentially avoidable investigations and procedures [1,24].

Current trauma pathways predominantly focus on structural repair and short-term analgesia. However, the reviewed evidence indicates that routine post-injury care should incorporate early risk stratification, screening for neuropathic and centralised pain features, and timely referral to multidisciplinary pain and rehabilitation services [2,21,23].

From a system perspective, investment in early multimodal pain management and psychologically informed rehabilitation may reduce long-term disability, opioid dependence, and health-care utilisation.

## 4. Discussion

### 4.1. Principal Findings

This scoping review demonstrates that persistent pain after orthopaedic trauma is common and frequently displays clinical features consistent with centrally mediated pain mechanisms. Across diverse trauma populations—including fracture surgery, pelvic injury, ankle fractures, and major blunt trauma—substantial proportions of patients developed long-term pain, neuropathic pain characteristics, psychological amplification, and functional disability that persisted well beyond the expected period of biological tissue healing [1,2,7,21,22,23]. These findings were observed consistently across prospective cohorts with follow-up ranging from months to several years, indicating that persistent post-traumatic pain is not an isolated or rare outcome but a frequent sequela of musculoskeletal injury.

Importantly, none of the included primary studies formally diagnosed central sensitisation using validated experimental criteria. Instead, this review synthesised evidence derived from CS-consistent proxy measures, including neuropathic pain descriptors, altered sensory symptoms, disproportionate pain persistence, psychological risk factors, and neurophysiological changes [2,12,21,22,23]. The convergence of these independent lines of evidence suggests that a clinically meaningful subset of trauma patients develops pain phenotypes compatible with centrally mediated mechanisms, even though definitive mechanistic confirmation was rarely performed.

The findings of this review do not support a model in which persistent post-traumatic pain is explained solely by residual tissue damage or incomplete structural recovery. Instead, pain trajectories after orthopaedic trauma appear heterogeneous and dynamic. Several cohorts demonstrated that neuropathic features and disproportionate pain can emerge or intensify over time despite fracture union and completion of standard rehabilitation [2,21]. When considered together, these findings align with broader pain literature showing that prolonged nociceptive input and psychological vulnerability can contribute to amplification of pain within the central nervous system [11,16,17,27].

Collectively, the primary evidence synthesised in this review indicates that persistent pain after orthopaedic trauma is multifactorial. Biological injury severity, peripheral nerve involvement, psychological vulnerability, and early pain intensity all appear to interact in shaping long-term outcomes [1,2,13,21,22,23]. Recognition of this heterogeneity is essential, as it suggests that structurally focused management strategies may be insufficient for some patients and that mechanism-informed assessment approaches are required within orthopaedic trauma care pathways.

### 4.2. Burden and Trajectories of Sensitisation-Consistent Pain After Orthopaedic Trauma

The primary studies included in this review collectively demonstrate that persistent pain following orthopaedic trauma is a frequent and clinically significant outcome. Across multiple cohorts, long-term pain was observed well beyond the period of expected structural healing [1,2,21,22,23]. Importantly, these pain outcomes were not uniform; rather, they followed variable trajectories, ranging from steady improvement to persistent or worsening symptoms over time.

A notable proportion of patients developed neuropathic pain characteristics during recovery, as identified through validated screening instruments [2,21,22,23]. The emergence of these features months after injury indicates that post-traumatic pain states can evolve dynamically rather than remaining fixed from the acute phase. This temporal pattern reflects a potential transition from predominantly nociceptive mechanisms toward more complex mixed or centrally influenced pain phenotypes [11,17,27].

Long-term follow-up studies further highlight the functional consequences of these trajectories. Persistent pain was consistently associated with reduced physical function, impaired participation in daily activities, and poorer quality of life [1,22,23,25]. Such findings emphasise that the impact of post-traumatic pain extends beyond symptom severity alone and has meaningful implications for rehabilitation and social recovery.

Together, these data indicate that sensitisation-consistent pain after orthopaedic trauma is prevalent, durable, and clinically consequential. These findings emphasise the need for early recognition of high-risk pain trajectories and for follow-up models that extend beyond confirmation of fracture union alone.

### 4.3. Mechanistic and Pathophysiological Interpretation

The clinical patterns identified in this review are biologically plausible within established models of pain neurophysiology. Acute fractures and soft tissue injuries generate intense and prolonged nociceptive inputs from bone, periosteum, and surrounding soft tissues capable of inducing spinal and supraspinal hyperexcitability, altering inhibitory control, and reorganising pain networks.

Experimental pain research has shown that sustained deep-tissue nociception facilitates temporal summation, receptive field expansion, referred pain, and widespread hyperalgesia [9,10,27]. Trauma, particularly when combined with surgery, prolonged inflammation, and immobilisation, provides a biological context that may facilitate this cascade, leading to disproportionate or persistent pain experiences in susceptible individuals [1,2,7,21,22,23]. Mechanistic reviews further describe alterations in descending inhibitory systems and imbalances between GABAergic and glutamatergic signalling as important contributors to the persistent pain state [16,17,20,26,29,30].

Preclinical fracture models further reinforce this biological trajectory from tissue injury to sustained central hyperexcitability. Contextual studies demonstrate that bone and soft-tissue injury can trigger prolonged neuroimmune activation, including microglial and chemokine-mediated responses, as well as activity-dependent synaptic plasticity within the dorsal horn [7,8,9,31,32]. Chemokine-mediated microglial activation, caspase-dependent synaptic plasticity, and changes in excitatory neurotransmission have been identified as the drivers of persistent fracture-related hyperalgesia [9,33,34]. These processes are like those observed in established nociplastic pain conditions and suggest that traumatic injury may act as a catalyst for central nervous system adaptations that outlast peripheral tissue repair [14,15,26,35,36]. Importantly, comparative syntheses indicate that the inflammatory and neuroimmune responses following traumatic injury are typically more heterogeneous and dysregulated than those observed after elective surgery, potentially increasing the likelihood of maladaptive plasticity [19,25,37,38].

**Figure 2:**
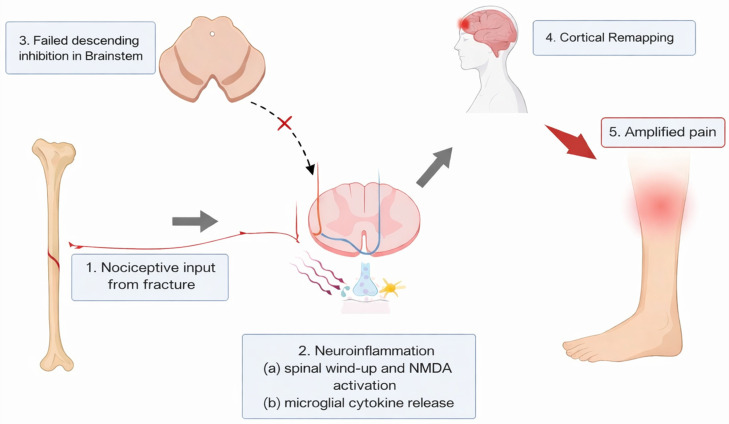
Central sensitisation mechanisms in orthopaedic trauma.

Direct measurement of central mechanisms in clinical trauma cohorts remains limited. However, neurophysiological alterations have been demonstrated during the acute post-fracture period in at least one included study using transcranial magnetic stimulation [12]. Although such data are sparse, they support the general concept that central processing changes can arise early after injury and need not be restricted to established chronic pain. Taken together, these experimental and translational findings provide a credible mechanistic framework for interpreting the CS-consistent clinical features observed in trauma populations, while acknowledging that such mechanisms are unlikely to account for all cases of persistent post-traumatic pain [14,15,25,26,33]. Figure 2 illustrates a conceptual model of the CS mechanisms after orthopaedic trauma.

### 4.4. Trauma Versus Elective Surgery

Orthopaedic trauma differs fundamentally from elective surgery in both the biological and clinical contexts. Traumatic injury is characterised by uncontrolled tissue destruction, variable degrees of soft-tissue and neural damage, and unpredictable inflammatory responses, whereas elective surgery involves planned and standardised operative insult with structured perioperative care. These distinctions are important when considering the risk of persistent pain and CS.

Trauma-specific syntheses emphasise that inflammatory and neuroimmune responses following unplanned injury are typically more dysregulated and heterogeneous than those observed after elective procedures [8,11,15]. Elective orthopaedic surgery occurs in a controlled environment with predictable tissue handling and established multimodal analgesic protocols, which generally result in narrower and more modifiable pain trajectories [19]. In contrast, trauma often involves higher nociceptive burden, prolonged immobilisation, and greater psychological and physiological stress, factors that are repeatedly identified as contributors to pain chronification after musculoskeletal injury [25,26].

This distinction supports the concept that orthopaedic trauma represents a uniquely high-risk context for the development of CS compared with routine elective orthopaedic procedures.

### 4.5. Clinical Phenotypes and Diagnostic Features

Clinical presentations of pain after orthopaedic trauma are heterogeneous and rarely reflect a single mechanism. Across the included cohorts, many patients reported persistent or disproportionate pain, sensory amplification, and functional limitation that could not be fully explained by radiological healing or surgical success [1,2,15]. Symptoms commonly fluctuate with physical load, sleep quality, emotional distress, and fear avoidance, reflecting the dynamic interactions between peripheral inputs and central modulation [39,40]. Such patterns indicate that post-traumatic pain frequently represents a mixture of nociceptive, neuropathic, and centrally mediated processes rather than a purely peripheral condition.

The timing of symptom development provides further insight. Objective evidence from one cohort demonstrated early neurophysiological alterations shortly after fracture [12], reinforcing the plausibility that central mechanisms may contribute from the acute phase. Consistent with this, severe early postoperative pain after orthopaedic trauma surgery is a strong predictor of persistent pain at 12 months, suggesting that adverse sensitisation trajectories can be established during initial recovery [13]. In several studies, neuropathic pain features emerged or intensified during follow-up rather than being confined to the immediate post-injury period [2,21,23]. This evolution supports the concept that pain phenotypes after trauma can shift over time as central modulation and psychological factors interact with ongoing peripheral inputs.

Clinical features commonly associated with CS, such as widespread pain, allodynia, sleep disturbance, and pain disproportionate to mechanical findings, have been reported in broader musculoskeletal populations [16,17,37,38]. However, within trauma cohorts, these features occur alongside clear structural injury, making mechanism attribution challenging. Functional outcomes are also closely linked to pain phenotype; patients with neuropathic or complex pain characteristics consistently demonstrate greater disability and poorer quality of life [1,2,23,24].

Overall, the available evidence suggests that trauma-related pain should be conceptualised as a dynamic clinical syndrome influenced by biological injury, neural sensitivity, and psychosocial context, rather than as a uniform postoperative complication.

### 4.6. Neuropathic Pain as a Practical Clinical Marker of Central Sensitisation

Within orthopaedic trauma populations, neuropathic pain features provide a clinically accessible indicator of sensitisation-consistent pain states. Across several cohorts, 20–40% of patients screened positive for neuropathic pain characteristics using validated tools such as DN4 or painDETECT following fracture surgery and other traumatic injuries [2,21,22,23]. These patients consistently reported greater pain intensity, higher levels of functional impairment, and poorer health-related quality of life than those without neuropathic features, indicating that pain phenotype is closely linked to outcome.

Importantly, the presence of neuropathic pain characteristics in trauma populations does not necessarily imply isolated peripheral nerve injury. In longitudinal studies, neuropathic features frequently developed or worsened during follow-up rather than being present immediately after injury [32,41,42,43]. This temporal pattern suggests that such symptoms may arise from evolving central mechanisms in addition to peripheral pathology. The overlap between neuropathic descriptors and centrally mediated pain phenomena has been widely recognised in musculoskeletal pain literature, where altered central processing can generate neuropathic-like sensory experiences in the absence of demonstrable nerve damage [16,17,41,42].

From a practical perspective, neuropathic pain screening tools offer a feasible method for identifying patients at risk of persistent pain within routine trauma care. Although these instruments cannot definitively diagnose CS, they capture symptom patterns that are strongly associated with poorer functional recovery and greater health-care utilisation [1,2,21,22,23]. Their use therefore provides a pragmatic approach to risk stratification and to the early identification of individuals who may benefit from mechanism-informed management strategies.

Overall, the consistent association between neuropathic pain characteristics and adverse outcomes supports their role as a useful clinical marker of complex, sensitisation-consistent pain after orthopaedic trauma. Incorporating such screening into follow-up pathways may help clinicians recognise patients for whom structurally focused interventions alone are unlikely to be sufficient.

### 4.7. Measurement and Diagnostic Limitations

Accurate identification of CS after orthopaedic trauma remains challenging because no validated trauma-specific diagnostic framework currently exists. Most instruments used to infer central pain mechanisms were originally developed for chronic musculoskeletal conditions and have uncertain validity when applied to heterogeneous trauma populations.

Systematic evaluations of available tools highlight substantial methodological limitations. Middlebrook et al. demonstrated that commonly used measures of CS, including quantitative sensory testing (QST), temporal summation, conditioned pain modulation, and the Central Sensitisation Inventory (CSI), show wide measurement error and inconsistent reliability in musculoskeletal trauma settings [16]. Similarly, Georgopoulos et al. reported that although QST can detect sensory hypersensitivity, its ability to predict outcomes or distinguish central from peripheral mechanisms is variable, limiting its routine clinical utility [17]. These findings indicate that current measurement approaches are better suited to research contexts than to everyday trauma practice.

In trauma cohorts, neuropathic pain screening tools such as DN4 and painDETECT have been used as practical proxies for centrally mediated pain states [2,21]. However, these instruments were designed to detect neuropathic pain rather than CS specifically, and significant conceptual overlap complicates interpretation. Widespread pain has also been proposed as a marker of central mechanisms, yet population studies suggest that it is strongly influenced by psychological distress and somatic symptom burden, reducing its specificity as a diagnostic indicator [14,37,38].

Neurophysiological techniques may offer greater mechanistic specificity. Transcranial magnetic stimulation has demonstrated altered cortical excitability in acute fracture patients [12], but such methods are resource-intensive and lack normative trauma benchmarks. Emerging approaches, including wearable sensor data and ecological momentary assessment, may provide complementary insights into pain behaviour and recovery trajectories [34,39,42,43], but these remain largely untested in trauma populations.

Overall, the absence of a gold-standard diagnostic approach and the limited validity of existing tools constrain both research synthesis and clinical decision-making, contributing to the under-recognition of centrally mediated pain in routine trauma care. Development of reliable, trauma-specific assessment frameworks represents a key priority for future work.

### 4.8. Implications for Early Diagnosis, Reoperations, and Morbidity

Although no included trauma study directly evaluated whether early identification of CS reduces reoperation rates or morbidity, several clinically important patterns emerged from trauma evidence. Across cohorts, persistent post-traumatic pain and neuropathic features frequently occurred despite radiographic healing and completion of standard orthopaedic care, indicating that a proportion of patients develop pain states that are not driven by ongoing structural pathology [1,2,21,22,23].

In such patients, continued pain may prompt repeated imaging, injections, or surgical consultations to provide a mechanical explanation. However, trauma cohort data have demonstrated that neuropathic pain features, high early pain burden, and psychological vulnerability are associated with an increased risk of persistent pain and disability [2,13,21]. In the absence of structured screening for centrally mediated pain mechanisms, these individuals may be misclassified as having unresolved tissue pathology, which increases the likelihood of unnecessary or low-yield surgical interventions with potential implications for iatrogenic harm, prolonged recovery, and health-care utilisation.

Early recognition of CS-consistent phenotypes using neuropathic pain screening, pain trajectories, and psychological risk markers could allow clinicians to redirect patients toward nonsurgical management pathways, including neuropathic pain pharmacotherapy, psychologically informed physiotherapy, graded activity exposure, and pain education. Although prospective trials are required, this represents a biologically and clinically plausible strategy to reduce avoidable reoperations, limit morbidity, and improve long-term outcomes following orthopaedic trauma.

### 4.9. Interventions and Management Implications

The evidence synthesised in this review indicates that the early post-injury period represents a critical window in which adverse pain trajectories may be initiated or modified. In primary trauma cohorts, severe acute postoperative pain consistently predicted persistent pain and disability at later follow-up [13], while neurophysiological evidence demonstrates that alterations in central processing can occur shortly after fracture [12]. These findings suggest that early pain control and mechanism-informed management may influence long-term outcomes.

Historically, post-traumatic pain management has relied heavily on opioid-based strategies. Long-term cohort studies indicate that persistent pain after major trauma is common despite standard care, and concerns have been raised that inadequate multimodal approaches may contribute to pain chronification and opioid-related morbidity [1]. Contemporary perioperative pain frameworks emphasise opioid-sparing, multimodal analgesia tailored to the biological and psychological needs of individual patients [19]. Trauma-focused reviews similarly advocate early integration of biological, psychological, and rehabilitative strategies within routine care pathways [11].

Although no included study directly evaluated treatments targeting CS after trauma, several approaches are supported by indirect evidence. Neuromodulatory pharmacotherapies such as duloxetine and pregabalin are widely recommended for neuropathic and centrally mediated pain states [20], and psychologically informed rehabilitation, graded activity exposure, and pain education have demonstrated benefit in related musculoskeletal populations [39,40,44]. Importantly, psychological vulnerability strongly influences treatment response: higher catastrophising and distress are associated with greater acute pain and poorer long-term outcomes [13,21], while trauma-related stress symptoms are closely linked to features of CS [14,15].

Taken together, these findings support a shift from purely structurally focused care toward early, mechanism-based management for patients at risk of persistent pain. Prospective trials are required to determine whether targeted multimodal strategies can prevent or attenuate the development of centrally mediated pain after orthopaedic trauma.

### 4.10. Clinical Implications

The findings of this review have several practical implications for the care of patients after orthopaedic trauma. A substantial proportion of individuals develop persistent pain phenotypes characterised by neuropathic features, psychological amplification, and functional impairment that are not fully explained by structural pathology alone [1,2,7,21,22,23]. Recognition of these patterns is important because conventional strategies focused solely on fracture healing or mechanical correction may be insufficient for this subgroup.

Early identification of patients at risk for centrally mediated pain represents a key clinical priority. High acute pain intensity, the presence of neuropathic pain characteristics, psychological distress, and disproportionate disability consistently predict poorer long-term outcomes [12,13]. Incorporating routine screening for these factors into trauma follow-up could help clinicians recognise patients who are unlikely to recover with structurally focused care alone. In such individuals, repeated imaging, revision surgery, or escalation of opioid therapy is unlikely to provide meaningful benefit and may expose patients to unnecessary harm.

A mechanism-informed approach offers a more appropriate framework for management. Patients with CS-consistent phenotypes may benefit from neuromodulatory pharmacotherapy, orthopaedic manual therapy, psychologically informed rehabilitation, and multidisciplinary pain management rather than additional structural interventions [11,19,20,26,39,40,44]. Embedding such approaches within fracture clinics and rehabilitation pathways could improve patient stratification, guide treatment selection, and reduce progression to long-term disability. Overall, these findings support the integration of pain mechanism assessment into routine orthopaedic trauma care. Recognising that persistent post-traumatic pain is often multifactorial can help clinicians align treatment strategies with underlying processes, improving outcomes and reducing avoidable interventions. Table 3 summarises the early indicators and their associated downstream clinical consequences to support risk stratification and mechanism-informed care after orthopaedic trauma.

### 4.11. Strengths, Limitations, and Future Research Priorities

This scoping review integrates evidence from diverse orthopaedic trauma populations, including fracture surgery, pelvic injury, unstable ankle fractures, and major trauma cohorts, and synthesises clinical, neurophysiological, and psychosocial data to map features consistent with CS after injury [1,2,7,21,22,23]. A key strength is the deliberate focus on primary trauma cohorts rather than extrapolation from elective surgery or non-traumatic musculoskeletal conditions. By combining clinical, psychological, and limited neurophysiological data, the review provides a comprehensive overview of how persistent pain manifests and evolves in real-world trauma settings.

Several important limitations should be acknowledged. Direct experimental assessments of CS, such as quantitative sensory testing or conditioned pain modulation, have rarely been applied, with most studies relying on proxy indicators, including neuropathic pain questionnaires, psychological measures, or disability scores [16,17,41,45]. Outcome measures were heterogeneous, follow-up durations varied widely, and confounding factors such as injury severity, comorbidity, and baseline pain were inconsistently addressed, limiting causal inference. In addition, the predominance of observational study designs restricts conclusions regarding mechanisms and treatment effects.

These limitations define priorities for future research. There is a need for validated trauma-specific tools to identify CS and prospective longitudinal studies mapping neurobiological, sensory, and psychological changes from the acute post-injury period through recovery. Importantly, randomised trials are required to test whether early mechanism-based interventions such as multimodal analgesia, pain neuroscience education, psychologically informed rehabilitation, or neuromodulatory therapies can prevent or attenuate the development of centrally mediated pain. Finally, translation into clinical practice will require integration of CS screening and management frameworks into routine trauma pathways, fracture clinics, and rehabilitation services.

Addressing these gaps will enable more precise identification of patients at risk for centrally mediated pain and support the development of targeted strategies to improve long-term outcomes after orthopaedic trauma.

## 5. Conclusions

This scoping review demonstrates that persistent pain after orthopaedic trauma is common and frequently exhibits features consistent with centrally mediated pain mechanisms, including neuropathic pain, psychological amplification, early central nervous system disinhibition, and long-term functional impairment. Across diverse trauma cohorts, pain often persists well beyond structural healing, indicating that ongoing nociception alone does not fully explain post-traumatic pain outcomes. Although direct experimental assessment of CS remains limited in trauma research, the convergence of clinical, neurophysiological, and psychosocial evidence supports the central role of pain amplification mechanisms in a substantial subgroup of patients. Failure to recognise these mechanisms may contribute to misdirected investigations and treatment escalation in some patients. Future trauma research should prioritise the early identification of central sensitisation using standardised measures and evaluate mechanism-based, multidisciplinary interventions aimed at preventing long-term disability. Recognising centrally mediated pain mechanisms as important contributors to post-traumatic pain represents an important step towards more precise, effective, and patient-centred orthopaedic trauma care.

## Figures and Tables

**Table 1:** Characteristics of Included Trauma Cohorts.

Study	Country	Design	*n*	Trauma	Follow-Up	CS Construct	CS Measures	Pain Outcomes	Function/QoL	Psych	Adjusted Analyses
Keene 2021 [2]	UK	Multicentre RCT secondary analysis	1547 (DN4 933)	Lower-limb fractures	3, 6 m	Neuropathic phenotype	DN4, NRS	Neuropathic pain; NRS	DRI; EQ-5D-5L	–	ISS, baseline pain, age, sex
Aulenkamp 2022 [21]	Germany	Prospective cohort	82	Fracture osteosynthesis	Pre-op–12 m	CPSP/neuropathic	DN4i; painDETECT	CPSP; NP	BPI; EQ-5D	HADS	Not stated
Kolstadbraaten 2019 [1]	Norway	Retrospective cohort	68	Major blunt trauma	6 y	Acute sensitisation (conceptual)	Custom	Chronic pain	–	–	ISS-adjusted logistic
Gerbershagen 2010 [22]	Germany	Cross-sectional follow-up	69	Pelvic fractures	52 m	Pain chronicity	MPSS; painDETECT	PPP; NRS; CPGQ	ODI; Majeed; SF-12	HADS	Nerve injury vs. MPSS
Rbia 2017 [23]	Netherlands	Retrospective survey	271	Ankle ORIF	5.8 y	Neuropathic pain	DN4; MPQ	Persistent pain; NP	QoL/work	–	Multivariable
Castillo 2017 [24]	USA	Multicentre RCT	495	Extremity fractures	3–12 m	Persistent pain risk	painDETECT; BPI	Acute and chronic pain	SMFA; VR-12	PHQ-9; PCL	ITT; centre-adjusted
Edgley 2019 [13]	Australia	Prospective cohort	229	Trauma surgery	3 m	Psych–pain vulnerability	NRS; PCS; WHODAS	PPSP	WHODAS	K10; PCS	Multivariable
Jodoin 2020 [12]	Canada	Case–control neurophysiology	56 + 28	Upper-limb fractures	≤14 d	Cortical disinhibition	TMS (SICI; ICF)	NRS ≥ 4	DASH	–	Pain→SICI
Wynne-Jones 2006 [18]	UK	Prospective cohort	490	MVC	6 m	Widespread pain	Body manikin	New WP	SF-8	GHQ; SSC	Poisson-adjusted

Abbreviations: CS = central sensitisation; RCT = randomised controlled trial; DN4 = Douleur Neuropathique en 4 questions; NRS = numerical rating scale; DRI = Disability Rating Index; EQ-5D = EuroQol-5 Dimension; CPSP = chronic post-surgical pain; NP = neuropathic pain; BPI = Brief Pain Inventory; HADS = Hospital Anxiety and Depression Scale; ISS = Injury Severity Score; MPSS = Mainz Pain Staging System; PPP = post-traumatic pelvic pain; CPGQ = Chronic Pain Grade Questionnaire; ODI = Oswestry Disability Index; MPQ = McGill Pain Questionnaire; ORIF = open reduction and internal fixation; SMFA = Short Musculoskeletal Function Assessment; VR-12 = Veterans RAND 12-Item Health Survey; PHQ-9 = Patient Health Questionnaire-9; PCL = PTSD Checklist; PCS = Pain Catastrophizing Scale; WHODAS = World Health Organisation Disability Assessment Schedule; K10 = Kessler Psychological Distress Scale; TMS = transcranial magnetic stimulation; SICI = short-interval intracortical inhibition; ICF = intracortical facilitation; DASH = Disabilities of the Arm, Shoulder and Hand; SF-8 = Short-Form-8; GHQ = General Health Questionnaire; SSC = Somatic Symptoms Checklist; MVC = motor vehicle collision; ITT = intention-to-treat, PPSP = persistent post-surgical pain; DN4i = Douleur Neuropathique en 4 questions, interview version; EQ-5D-5L = EuroQol 5-Dimension 5-Level questionnaire; QoL = quality of life;WP = widespread pain.

**Table 2:** Synthesis of Pain Phenotypes Across Studies.

Study	Key Prevalence Signal	Predominant Phenotype Signals
Aulenkamp [21]	CPSP 57.1% (3 m), 42.7% (12 m); neuropathic CPSP 7.7% (3 m), 17.1% (12 m)	Mixed; neuropathic features in a meaningful minority (DN4i)
Edgley [13]	Persistent pain 65% (3 m)	Mixed; captures psychosocial risk and disability (not neuropathic-specific)
Keene [2]	Neuropathic pain 32% (3 m), 30% (6 m); non-neuropathic chronic pain 56% (3 m), 53% (6 m)	Neuropathic + non-neuropathic chronic pain both common (DN4)
Rbia [23]	Neuropathic pain symptoms 23%	Neuropathic symptoms prominent in the long-term subgroup (DN4 + MPQ)
Gerbershagen [22]	Pelvic pain 64%; dysfunctional chronicity stages common	Chronicity/impact phenotype; neuropathic association via painDETECT/nerve injury correlations
Kolstadbraaten [1]	Pain 69%; severe pain 24%	Chronic persistent pain burden; treatment-pattern signals (opioid-heavy early care)
Wynne-Jones [18]	New widespread pain 8%	Widespread pain phenotype; psychosocial confounding emphasised
Jodoin [12]	Group differences by pain intensity	Early central mechanism signal (cortical disinhibition/facilitation changes in moderate–severe pain)
Castillo [24]	Not a prevalence study	System-level focus on acute pain management and longer-term outcomes (pain/opioids/function/PTSD)

**Table 3:** Early indicators of centrally mediated pain risk and downstream clinical consequences after orthopaedic trauma.

Early Post-Trauma Indicators	Evidence from Trauma Cohorts	Associated Downstream Outcomes	Clinical Implications
Severe acute pain intensity	High early pain scores predict persistent pain and disability	Chronic pain, prolonged rehabilitation, opioid dependence	Early aggressive multimodal analgesia; early review
Neuropathic pain features (e.g., DN4, painDETECT)	Neuropathic descriptors common after fractures and polytrauma	Worse pain, poorer function, higher health-care use	Consider centrally mediated pain pathways
Early sensory hypersensitivity or widespread pain	Trauma linked to later widespread pain development	Diffuse pain syndromes, poor response to local treatment	Avoid repeated local interventions
Psychological distress (catastrophising, PTSD, depression)	Strongly associated with pain chronicity and disability	Treatment resistance, delayed recovery	Early psychological screening and support
High opioid exposure without multimodal strategies	Associated with persistent pain trajectories	Opioid dependence, poor pain control	Prioritise multimodal and non-opioid strategies
Neural injury (nerve damage, crush injury)	Higher risk of neuropathic and persistent pain	Long-term disability	Early pain specialist referral

## Data Availability

No new data were created or analysed in this study. All data supporting the findings of this scoping review are derived from publicly available published sources, which are cited within the manuscript and listed in the reference section.

## Supplementary Materials

The following supporting information can be downloaded at https://www.mdpi.com/article/10.3390/jcm15031035/s1. Table S1: Detailed characteristics and data extraction for all included sources of primary evidence. Table S2: Detailed characteristics and data extraction of all included sources of secondary evidence.

## Abbreviations

ACR—American College of Rheumatology; BPI—Brief Pain Inventory; CPGQ—Chronic Pain Grading Questionnaire; CPSP—Chronic Post-Surgical Pain; CS—Central Sensitisation; CSI—Central Sensitisation Inventory; DASH—Disabilities of the Arm, Shoulder and Hand; DN4—Douleur Neuropathique 4 Questions; DN4i—DN4 interview; DRI—Disability Rating Index; EQ-5D—EuroQol-5 Dimensions; EQ-5D-3L—EuroQol-5 Dimensions, three-level version; EQ-5D-5L—EuroQol-5 Dimensions, five-level version; GHQ—General Health Questionnaire; HADS—Hospital Anxiety and Depression Scale; HADS-A—Hospital Anxiety Scale; HADS-D—Hospital Depression Scale; ICF—Intracortical Facilitation; ISS—Injury Severity Score; K10—Kessler Psychological Distress Scale; LICI—Long-Interval Intracortical Inhibition; MPQ—McGill Pain Questionnaire; MPSS—Mainz Pain Staging System; MPOPS—Multidimensional Postoperative Pain Scale; MVC—Motor-Vehicle Collision; NRS—Numerical Rating Scale; ORIF—Open Reduction and Internal Fixation; PACU—Post-Anaesthesia Care Unit; PCS—Pain Catastrophizing Scale; PCL—PTSD Checklist; PHQ-9—Patient Health Questionnaire-9; PPP—Post-traumatic Pelvic Pain; PRISMA-ScR—Preferred Reporting Items for Systematic Reviews and Meta-Analyses Extension for Scoping Reviews; QST—Quantitative Sensory Testing; rMT—Resting Motor Threshold; RR—Relative Risk; SES—Subjective Experience of Sensation; SF-8—Short Form-8 Health Survey; SF-12—Short Form-12 Health Survey; SICI—Short-Interval Intracortical Inhibition; SMFA—Short Musculoskeletal Function Assessment; SSC—Somatic Symptoms Checklist; TMS—Transcranial Magnetic Stimulation; VAS—Visual Analogue Scale; VR-12—Veterans RAND 12-Item Health Survey; WHODAS—World Health Organisation Disability Assessment Schedule; WHiST—Wound Healing in Surgical Trauma Trial; WP—Widespread Pain; PPSP—Persistent Post-Surgical Pain; QoL—Quality of Life.
