# Supplementary material for: Central Sensitisation After Orthopaedic Trauma: An Overlooked Contributor to Chronic Pain and Functional Disability—A Scoping Review
Source: J Clin Med. 2026 Jan 28;15(3):1035. doi: 10.3390/jcm15031035 (PMC12898706; doi:10.3390/jcm15031035)
Supplement: Supplementary file 1 [file jcm-15-01035-s001.zip › jcm-4097067-supplementary.pdf]

Supplementary Table S1. Detailed Characteristics of Primary Evidence Included in the Review

Psych/PTSD measures	Analyses
Not reported	Regression adjusted incl. ISS, baseline scores, age/sex, stratification factors
HADS (anxiety/depression)	Descriptive comparisons: adjustment not stated
Not assessed (explicitly)	Logistic regression adjusted incl. ISS and other variables; baseline pain not adjusted
HADS-Anxiety; HADS-Depression	Nerve injury associated with a higher chronicity stage (analysis details not fully reported)
Not reported	Multivariable adjustment (age, hypertension, dislocation, late complications); baseline pain not stated
PHQ-9 depression; PTSD Checklist (PCL) at 12 months	Intention-to-treat RCT; regression modelling planned to adjust for baseline covariates; hierarchical models for centre effects
Kessler-10 psychological distress; PCS catastrophising	Multivariable logistic regression: female sex & prior post-injury surgery → severe acute pain; pre-op NRS → persistent pain
Not reported	Linear regression: pain intensity (not time since injury) predicted SICI and ICF abnormalities
GHQ (psychological distress); Somatic Symptoms Checklist	Poisson regression adjusting for age, sex, and psychological distress & somatic symptoms

Pain outcomes	Function/QoL outcomes
Neuropathic pain prevalence; pain severity (NRS 0–10)	DRI; EQ-5D-5L
CPSP incidence at 3/12 m; neuropathic CPSP at 3/12 m; NRS rest/movement	BPI (functional impairment); EQ-5D-3L/VAS
Chronic pain; " severe chronic pain" (defined by frequency/intensity)	Not assessed (explicitly)
Post-traumatic pelvic pain (PPP) prevalence; NRS average & worst pain; Chronic Pain Grading Questionnaire	Oswestry Disability Index; Majeed score; Bürk score; GST score; SF-12
Prevalence/pattern of persistent pain; neuropathic pain prevalence (23%)	QoL/work impact (custom items)
Acute and chronic pain intensity; opioid consumption; persistent pain at 3/6/12 months	Short Musculoskeletal Function Assessment (SMFA); VR-12 health survey
Severe acute PACU pain (NRS ≥ 6); persistent post-surgical pain at 3 months	WHODAS (disability threshold ≥25%)
NRS current pain; moderate–severe pain defined as NRS ≥ 4	DASH disability score
New-onset widespread pain at 6 months (8% crash vs 4% non-crash)	SF-8 Health Survey (mental & physical health)

**CS-related measure(s)**
DN4 (≥3/7), NRS pain
DN4 interview (DN4i); painDETECT; SES (pain perception)
Custom questionnaire
MPSS (stages I–III); painDETECT (neuropathic pain); pain distribution
DN4 (≥4/10); MPQ; VAS pain
painDETECT; Brief Pain Inventory (BPI); Multidimensional Post-operative Pain Scale (MPOPS)
NRS pain, modified Brief Pain Inventory-SF, Pain Catastrophizing Scale (PCS), WHODAS 2.0, Kessler-10 distress
TMS: SICI (GABA-A inhibition), ICF (glutamatergic facilitation), LICI (GABA-B), rMT
Whole-body pain manikins; ACR widespread pain definition

Time since injury/assessment	CS construct/definition used
3 and 6 months	No explicit CS definition; focuses on neuropathic characteristics
Pre-op; days 1–5; 6 w; 3 m; 1 y	No explicit CS measure; CPSP/neuropathic CPSP focus
6 years	Mentions acute sensitisation conceptually; no operational CS definition
Median follow-up 52 months	Not framed as CS; evaluates chronicity/neuropathic traits
Mean 5.8±1.9 years	No CS: neuropathic pain defined per IASP
Enroll ≤48h–10d; follow-up 3/6/12 m	No formal CS label: persistent pain and neuropathic pain is conceptualised as a risk for chronic centralised pain
Pre-op; PACU: 72h post-op; 3 months	No CS; focuses on persistent post-surgical pain & psychological factors
Within 14 days post-trauma (mean 4.5–7.6 days depending on pain group)	Central vulnerability inferred from cortical excitability change
Baseline <7 days; follow-up 6 months	Widespread pain (ACR fibromyalgia criteria) as a proxy of centralised pain

**Population (n)**	**Trauma type / region**
Trial 1,547; DN4 available ≥1 timepoint in 933	Post-op lower-limb major trauma (acetabulum/femur; patella/tibia/fibula/foot)
Enrolled 127; analysed 82 at 1 year	Traumatic fractures requiring osteosynthesis (extremities 91.3%)
68 patients	Moderate–severe blunt trauma (ISS ≥9)
69 patients	Pelvic ring and acetabular fractures
271	Unstable ankle fractures treated with ORIF
495 planned (165/arm)	Extremity fractures (upper/lower, multiple sites listed)
303 assessed for acute pain; 229 followed up to 3 months	Orthopaedic trauma surgery population (index injury/operation)
56 isolated upper-limb fracture (IULF) patients; 28 healthy controls	Isolated upper-limb fractures
597 baseline respondents; 490 with complete 6-month pain data	Motor-vehicle crash (all body regions; not restricted to a specific anatomical site)

First author (year)	Country/setting	Design
**Keene (2021)**	UK, 24 major trauma centres	Secondary analysis of multicentre RCT dataset (WHiST)
**Aulenkamp (2022)**	Germany, single centre (Ruhr University Bochum)	Prospective observational
**Kolstadbraaten (2019)**	Norway, single trauma centre (Oslo University Hospital)	Observational follow-up (retrospective)
**Gerbershagen (2010)**	Germany – University of Cologne trauma centre	Cross-sectional follow-up of trauma cohort
**Rbia (2017)**	Netherlands, teaching hospital	Retrospective survey
**Castillo (2017)**	USA, 21 trauma centres (METRC)	Multicentre RCT protocol
**Edgley (2019)**	Australia – Royal Melbourne Hospital	Prospective observational cohort
**Jodoin (2020)**	Canada (Level 1 trauma centre)	Prospective case–control neurophysiology study
**Wynne-Jones (2006)**	UK, national motor insurance cohort	Prospective cohort

Supplementary Table S2. Detailed Characteristics of Secondary Evidence Included in the Review

A. Systematic Reviews and Meta-Analyses (n = 4)

**Study (PDF)**	**Type of Review**	**Population/Scope**	**Main Methods**	**Key Findings Relevant to CS After Trauma**	**How Used in the Scoping Review**
**Middlebrook et al., 2020**	Systematic review	Musculoskeletal trauma populations	Reviewed measurement properties of CS-related tools (CSI, QST, DN4, painDETECT)	Demonstrated that most CS tools lack trauma-specific validation; psychometric performance variable	Provided methodological framework for interpreting assessment tools
**Georgopoulos et al., 2019**	Systematic review & meta-analysis	Chronic MSK pain cohorts	Meta-analysis of quantitative sensory testing as predictor of outcomes	Abnormal sensory thresholds predict persistent pain and disability	Used to interpret sensory testing as proxy markers of CS
**Alkassabi et al., 2022**	Systematic review	Musculoskeletal injuries	Synthesis of prognostic factors for persistent pain	Early pain intensity, psychological distress, and neuropathic features are consistent predictors	Provided prognostic context for trauma cohorts

**Rosenbloom et al., 2013**	Systematic review	Traumatic MSK injuries	Review of incidence of persistent pain and psychological outcomes	Persistent pain common up to 7 years; strong association with anxiety, depression, PTSD	Contextualised long-term pain trajectories after trauma

B. Narrative and Consensus Reviews (n = 6)

Study (PDF)	Focus	Evidence Base	Mechanisms Described	Relevance to Trauma CS
**Nishimura et al., 2025**	Fracture pain mechanisms	Translational review (clinical + preclinical)	Peripheral sensitisation, NGF/TrkA signaling, central sensitisation after fracture	Direct biological rationale for CS after bone injury
**Zhao et al., 2022**	Chronic pain after bone fracture	Molecular and therapeutic review	Microglial activation, AMPA/NMDA plasticity, neuroinflammation	Provides mechanistic pathways linking fracture to CS
**Hartman Budnik et al., 2025**	OTA consensus symposium	Expert consensus	New conceptual models of post-traumatic pain	Framed trauma-specific paradigm for centralised pain
**Karateev et al., 2022**	Chronic post-traumatic pain	Narrative review	Multifactorial pathogenesis (neural injury, inflammation, CS)	Integrated rheumatologic and orthopaedic perspectives
**Chunduri & Aggarwal, 2022**	Multimodal perioperative pain	Clinical review	Opioid-sparing, regional techniques	Clinical implications of mechanism-based management
**Arendt-Nielsen et al., 2011**	Transition from acute to chronic MSK pain	Experimental pain science review	Temporal summation, descending modulation, hyperalgesia	Provided foundational CS biology applicable to trauma

C. Contextual Clinical / Mechanistic Studies (n = 2)

Study (PDF)	Design	Population	Core Findings	Contribution
**Manuel et al., 2023**	Observational study	Chronic pain patients with PTSD exposure	Strong association between traumatic stress and CS features	Demonstrated psychological amplification of CS
**Langeveld et al., 2025**	Clinical review	Post-amputation pain	High prevalence of neuropathic and centralised pain mechanisms	Example of trauma-induced centralised pain states
